# Genetic components of *Escherichia coli* involved in its complex prey-predator interaction with *Myxococcus xanthus*

**DOI:** 10.3389/fmicb.2023.1304874

**Published:** 2023-12-05

**Authors:** Ning Zhang, Tingyi Li, Hongwei Pan, Yipeng Wang, Qi Li, Jia Luan, Xuesong He, Wenyuan Shi, Yuezhong Li, Chuandong Wang, Fengyu Zhang, Wei Hu

**Affiliations:** ^1^State Key Laboratory of Microbial Technology, Microbial Technology Institute, Shandong University, Qingdao, Shandong, China; ^2^Department of Clinical Laboratory, Qilu Hospital of Shandong University, Jinan, Shandong, China; ^3^Immunology and Molecular Genetics, University of California, Los Angeles, Los Angeles, CA, United States; ^4^Department of Microbiology, The Forsyth Institute, Cambridge, MA, United States; ^5^Department of Oral Medicine, Infection and Immunity, Harvard School of Dental Medicine, Boston, MA, United States

**Keywords:** *Myxococcus xanthus*, *Escherichia coli*, predator-prey interaction, *fis* gene, *dusB* gene, flagellum, myxovirescin A

## Abstract

*Myxococcus xanthus* and *Escherichia coli* represent a well-studied microbial predator-prey pair frequently examined in laboratory settings. While significant progress has been made in comprehending the mechanisms governing *M. xanthus* predation, various aspects of the response and defensive mechanisms of *E. coli* as prey remain elusive. In this study, the *E. coli* MG1655 large-scale chromosome deletion library was screened, and a mutant designated as ME5012 was identified to possess significantly reduced susceptibility to predation by *M. xanthus*. Within the deleted region of ME5012 encompassing seven genes, the significance of *dusB* and *fis* genes in driving the observed phenotype became apparent. Specifically, the deletion of *fis* resulted in a notable reduction in flagellum production in *E. coli*, contributing to a certain level of resistance against predation by *M. xanthus*. Meanwhile, the removal of *dusB* in *E. coli* led to diminished inducibility of myxovirescin A production by *M. xanthus*, accompanied by a slight decrease in susceptibility to myxovirescin A. These findings shed light on the molecular mechanisms underlying the complex interaction between *M. xanthus* and *E. coli* in a predatory context.

## Introduction

Predatory bacteria are pervasive in the natural environment, employing diverse strategies to prey upon various microorganisms and exerting significant influence on the modulation of microbial population structures and dynamics (Hungate et al., 2021). Notably, *Myxococcus xanthus* stands out as a well-documented predatory species, characterized by a complex epibiotic predation strategy (Perez et al., 2016; Thiery and Kaimer, 2020; Zwarycz et al., 2023), which infers a wide-ranging prey spectrum encompassing gram-positive and gram-negative bacteria (Livingstone et al., 2017; Arend et al., 2021), as well as certain fungi (Lloyd and Whitworth, 2017). The predation mechanisms employed by *M. xanthus* are multifaceted, operating either independently or synergistically to kill and consume different preys (Thiery and Kaimer, 2020; Sydney et al., 2021; Kaimer et al., 2023). *M. xanthus* cells search and encounter the prey by slowly gliding over surfaces powered by two motility mechanisms (Munoz-Dorado et al., 2016; Rombouts et al., 2023); a single predator cell approaches and dispatches a prey cell in a contact-dependent manner (Zhang W. et al., 2020), involving the concerted action of a tad-like apparatus (Seef et al., 2021) and a type III-like system (T3SS*) (Thiery et al., 2022); the secondary metabolites (Xiao et al., 2011), such as myxovirescin A (also known as antibiotic TA), and secreted hydrolytic enzymes like protease MepA (Berleman et al., 2014), are vital components of the predation arsenal, effectively targeting prey cells with the assistance of outer membrane vesicles (OMVs) (Zwarycz et al., 2020). These intricate procedures necessitate the participation of either individuals or a substantial population of predator cells, potentially eliciting a distinct reaction from the prey (Hungate et al., 2021).

In the context of predatory dynamics, prey organisms may employ defensive strategies in response to the predatory actions of *M. xanthus* (Muller et al., 2014; Perez et al., 2014; Sydney et al., 2021). *Escherichia coli* is one of the most commonly studied prey organisms killed by *M. xanthus* (DePas et al., 2014; Livingstone et al., 2018; Sydney et al., 2021). The development of biofilms, specifically the production of the curli and cellulose matrix, serves as a protective barrier for *E. coli* against the predation efforts of *M. xanthus* (DePas et al., 2014). Reciprocal adaptations have been observed in an artificial evolution experiment involving predator-prey co-cultures of *M. xanthus* and *E. coli*, resulting in the induction of outer-membrane protease OmpT aggregations and mucoidy development in *E. coli* as defensive responses (Nair et al., 2019). The overexpression of *lspA*, encoding the bacterial type II signal peptidase (SPase II), enhances TA resistance in *E. coli* and presents a potential defense mechanism against *M. xanthus* (Xiao and Wall, 2014). Additionally, transcriptomic analysis has revealed that nearly 40% of *E. coli* genes exhibit significant differential expression when co-incubated with *M. xanthus* (Livingstone et al., 2018). Despite the significant advancements in understanding the fundamental mechanisms underlying *M. xanthus* predation and *E. coli* defense, many facets of their interaction remain enigmatic.

In this study, we conducted a systematic screening of the *E. coli* Large-scale Chromosome Deletion Library (National Bioresource Project, NBRP) to reveal potential molecular constituents of *E. coli* involved in its predator-prey interaction with *M. xanthus* as a prey organism. Leveraging the well-established genetic systems of these microorganisms and employing a double-layer agar assay, we identified novel genetic elements within *E. coli* that play a role in its defense against *M. xanthus* predation. The findings from this research provide some insights into the underlying molecular mechanisms of this complex predator-prey interaction.

## Materials and methods

### Bacterial strains and cultural condition

The bacterial strains used in this study are listed in the Supplementary Table 1. All the *M. xanthus* strains were cultured in CYE liquid medium (pH 7.6) or on CYE plates supplemented with 1.5% agar at 30°C (Campos et al., 1978). *E. coli* cells were cultured in liquid Luria-Bertani (LB) medium or M9 medium at 37°C (Zhang F. et al., 2020). A total of 124 ME strains from the *E. coli* Large-scale Chromosome Deletion Library (Kato and Hashimoto, 2008) at the Japan National Institute of Genetics were acquired from Dr. Barry Warner and cultivated following the provided instructions. When needed, kanamycin (Km, 40 μg/mL), ampicillin (Amp, 100 μg/mL), chloramphenicol (Chl 17 μg/mL), or spectinomycin (Spe, 30 μg/mL) was added to the medium.

### Double-layered agar assay

The assay was derived from prior studies on bacterial-phage interactions (Moce-Llivina et al., 2004; Santos et al., 2009). Briefly, 5 μL aliquot of *M. xanthus* cell suspension (OD_600__*nm*_ = 10) was spotted on CYE plates with 3–5 replicates, incubated at 30°C for 24 h, and 200 μL aliquot of overnight cultured *E. coli* (OD_600__*nm*_ = 4) was mixed with 3 mL 0.3% CYE soft agar and overlaid onto CYE plates with *M. xanthus* spots. Comparing reduced inhibition zones around *M. xanthus* spots were assessed to determine predation efficacy after another 24 h co-incubation. For screening, all *E. coli* mutants were sourced from the *E. coli* Large-scale Chromosome Deletion Library of NBRP, stored in the liquid nitrogen, and subsequently re-cultured in LB medium.

### Colony-invasion predation assay

Quantified colony-invasion predation assay was conducted as previously described with some modifications (Berleman and Kirby, 2007; Wang et al., 2019). *M. xanthus* and *E. coli* cells were harvested to log phase, and concentrated to OD_600__*nm*_ as 35 and 100 with 10 mM MOPS buffer (pH 7.6), respectively. On CFL medium with 1.5% agar, 2.5 μL of *E. coli* suspension was pipetted at a distance of 5 mm from 2.5 μL of *M. xanthus* spots with the assistance of the microscope (Eclipse E400, Nikon, Japan). Plates were incubated at 30°C, and predator-prey colonies were documented and collected for quantification at timescales. The viable *E. coli* cells were quantified through the counting of colony-forming units (CFU) on LB plates by the automatic colony analyzer (Czone G6T, Shineso, China). A minimum of biological triplicates was conducted to ensure accuracy.

### Construction of *E. coli* mutants and complement strains

*Escherichia coli* gene knockout mutants, including in-frame deletion of every gene encompassed by ME5012, Δ*flhDC*, and Δ*tolC*, were obtained based on the λ-Red recombinase-mediated system assay as previously described (Datsenko and Wanner, 2000; Tas et al., 2015; Zhang F. et al., 2020). The primers and plasmids were detailed in the Supplementary Table 1. Kanamycin gene cassettes, bordered by FRT (FLP recognition target) sites, and homologous arms were amplified through polymerase chain reaction (PCR) with templates of plasmid pKD4 or MG1655 DNA, respectively. These amplified sequences were assembled into a single sequence and electroporated into *E. coli* MG1655 harboring plasmid pTKRed for target-gene replacement. Gene deletion mutants were identified through a 2 × T5 Super PCR Mix kit (Tsingke, China), and the resistance marker was further excised through plasmid pCP20 at 42°C.

The p*fis*, p*dusB* and p*dusB*-*fis* plasmids were constructed using Gibson assembly method (Gibson et al., 2009), and detailed information is provided in Supplementary Table 1. The linearized plasmid vector and their genes containing homologous arms were amplified using pTrc99a or *E. coli* MG1655 genome as templates through PCR using corresponding primers (Supplementary Table 1). The construction of expression plasmids followed the guidelines of the Gibson assembly kit (New England Biolabs, China). Positive clones were subsequently verified through DNA sequencing. The plasmids were individually introduced into MG1655 Δ*fis*, Δ*dusB* or Δ*dusB-fis* strains to construct complement strains via chemical transformation.

### iTRAQ quantitative proteomic analysis

ME5012 and MG1655 were delivered with three replicates for quantitative proteomic analysis through the isobaric tags for relative and absolute quantitation (iTRAQ) methodology (Wang et al., 2016). Initially, cells were lysed to extract proteins, and they were reduced into peptides through trypsin digestion. Peptides sequentially were labeled individually with iTRAQ reagents for 2 h at room temperature. These labeled peptides were pooled and fractionated through strong cation exchange fractionation on high performance liquid chromatography (HPLC, LC-20AB, Shimadzu, Japan). After drying under vacuum, the labeled samples were analyzed using liquid chromatography-tandem mass spectrometry (LC-MS/MS, Thermo Fisher, USA).

The raw MS data were processed and analyzed to identify differentially expressed proteins (DEPs) for mechanisms exploration as previously described (Jeong et al., 2001; von Mering et al., 2005). The statistically significant difference of a protein was considered with fold change (FC) >1.5 in the expression level and FDR *q*-value < 0.05. The pathway enrichment analysis was obtained based on the Kyoto Encyclopedia of Genes and Genomes (KEGG) database, and top KEGG enrichment results were listed with *p*-value < 0.05. Protein-protein interaction (PPI) network of DEPs was predicted through String database and was further visualized based on Degree Centrality Analysis using Cytoscape software 3.10.0 (Majeed and Mukhtar, 2023). The mass spectrometry proteomics data have been deposited to the ProteomeXchange Consortium via the PRIDE partner repository with the dataset identifier PXD046017.

### TEM observation

Morphologies of *E. coli* mutants were examined using a transmission electron microscopy (TEM) assay (Cujova et al., 2013). Briefly, *E. coli* mutants were absorbed on a carbon-stabilized copper grid for 5 min and subsequentially negative stained with 0.25% aqueous phosphotungstic acid for 10 s. Observations were performed through a STEM microscope (Talos F200X, Thermo Fisher).

### Motility assay

The motility assay was conducted as previously described (Teng et al., 2010). *E. coli* mutants were grown in the LB medium and allowed to incubate overnight. A total of 0.5 μL of cell suspension (OD_600_ = 10.0) was spotted onto 0.2% agar LB plates and incubated at 37°C for 5 h. Motility halos were documented and measured by a Czone G6T automatic colony analyzer.

### Bacterial tracking and analysis

Sample preparation was as previously described (Zhang W. et al., 2020; Thiery et al., 2022). *M. xanthus* DK1622 and *E. coli* were harvested and mixed at a ratio of 1:10, the mixture (1 μL) was applied to object slides with 1% CFL agar, overlaid with a cover glass, and incubated for 10 min at room temperature. The videos of predation were taken through the BX51 microscope equipped with a 100 × oil objective (Olympus, Japan). Bright-field images, with the dimensions of 133.2 μm × 88.8 μm, were acquired every 2 s, over a period of about 2 h. The bacterial movements were tracked and quantitative analyzed as previously described (Hu et al., 2016; Zhang W. et al., 2020). The primary steps encompass image background subtraction, smoothing processing, binarization processing, bacterial identification, and geometric information extraction. By evaluating kinematic parameters such as mean speed distribution, the rate of approaching prey, and the speed of leaving, the quantitative assessment of the alterations in speed during the predation process of the DK1622 cells was performed.

### Real-time quantitative PCR (RT-qPCR)

The RT-qPCR procedure was carried out based on the published methodology (Zhang F. et al., 2020). In brief, total RNA was extracted following the instructions of the MiniBEST universal RNA extraction kit (TaKaRa, China), and qualified by NanoDrop 2000 UV-vis spectrophotometer (Thermo Fisher). cDNA synthesis was conducted by the 5 × HiScript III qRT SuperMix (Vazyme, China). The allocation system of samples was meticulously prepared to employ 2 × AceQ Universal SYBR qPCR Master Mix (Vazyme). Related primers utilized in this process have been cataloged in the Supplementary Table 1. The RT-qPCR results were quantified using Quant Studio Design and Analysis Software v1.3.1 (Thermo Fisher). The *gapA* expression was set as the reference gene to normalize the target gene expression (Li et al., 2017).

### MIC measurement

The minimal inhibitory concentrations (MICs) of *E. coli* toward TA were determined following the guidelines from EUCAST (Kassim et al., 2016; Cunrath et al., 2019). The TA was prepared and purified from the fermentative broth of *M. xanthus* DK1622 as previously described (Wang et al., 2019).

### Antibiotic TA quantification

The extraction and detection of TA was performed as described previously described (Simunovic et al., 2006; Wang et al., 2019). Extracted fractions that contained TA were detected by HPLC (LC-20AD) with XTerra MS C18 column (5 μm, 2.1 mm × 100 mm, Waters, USA). The test conditions were set as: flow rate is 0.3 mL/min, solvent A is formic acid water solution (0.1%), solvent B is acetonitrile with formic acid (0.1%), and the elution program is 0–2 min with 5% B, 2–10 min to 50% B, 10–27 min to 95% B, 27–40 min maintaining 95% B. TA showed a characteristic UV_*max*_ absorption at 239 nm and was further confirmed by its mass spectrum. The exact amount of TA was calculated using the relative peak areas produced by the injection of a known amount of standard TA compound.

### Efflux pump activity assay

Efflux pump activity was determined by a Nile Red assay with minor modifications (Bohnert et al., 2010; Cunrath et al., 2019). After an overnight incubation within M9 medium at 37°C, *E. coli* cells were collected and washed with PPB buffer, treated with 10 μM carbonyl cyanide m-chlorophenylhydrazone (CCCP) for 15 min. A total of 10 μL 5 mM Nile Red solution was sequentially added, and incubated for 3 h at 37°C, and the mixed samples were rested at room temperature for 1 h. Cells were collected by centrifugation and resuspended with PPB buffer, and 200 μL of samples were transferred to a 96-well plate and measured fluorescence (excitation at 552 nm and emission at 636 nm) for 120 s, then 1 μL of 1 M glucose was added, and the signal was recorded for additional 300 s. The percentage fluorescence decreases and efflux half-time after energization of *E. coli* strains were calculated as previously described (Cunrath et al., 2019).

### Statistical analysis

Three biological replicates were conducted for quantitative analysis unless indicated otherwise. The results were expressed as mean ± SD, and One-way ANOVA analysis was employed for group comparisons. Visualization of results was accomplished by GraphPad Prism 9 software (La Jolla, CA, USA) and R packages (Li et al., 2023).

## Results

### The screening of an *E. coli* large-scale chromosome deletion mutant library to identify its genetic components involved in the predator-prey interaction with *M. xanthus*

**FIGURE 1:**
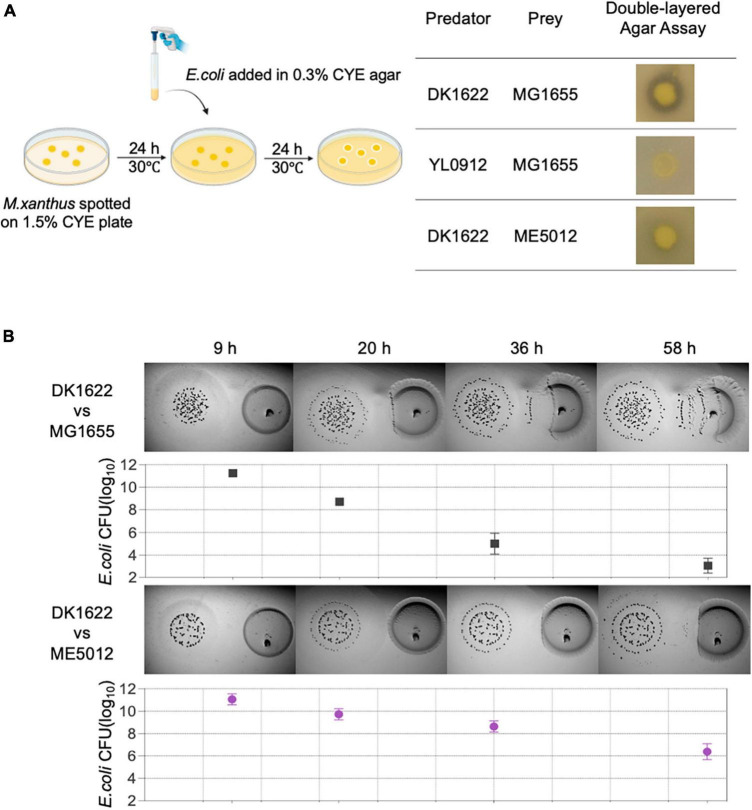
The predation efficiency of *M. xanthus* DK1622 on *E. coli* mutant ME5012 compared to wild-type MG1655. **(A)** Left illustration depicts the double-layered agar assay used to assess predator-prey interspecies interaction between *M. xanthus* and *E. coli*, and the representative screening results are shown in the right panel. **(B)** Visualization of colony invasion of *M. xanthus* DK1622 (left) into *E. coli* MG1655 and ME5012 (right), with the distance between the two colonies set at 5 mm. The surviving *E. coli* cells were quantified by CFU determination at set intervals.

A double-layer agar assay (Figure 1A-right panel) adapted from prior studies on bacterium-phage interactions (Cormier and Janes, 2014) was employed as an initial screening method to assess the predation efficacy of *M. xanthus* on various *E. coli* strains. To execute this assay, overnight cultured *E. coli* cells were amalgamated with semi-hard CYE agar, which was subsequently poured onto a CYE solid plate that had been previously incubated with *M. xanthus* colonies for a duration of 24 h. The wild-type (WT) *M. xanthus* DK1622 exhibited a clear zone of killing around its colonies after an overnight incubation period (Figure 1A-left panel), indicating its capacity for positive predation against WT *E. coli* MG1655. Conversely, *M. xanthus* YL0912 mutant deficient in TA production (Δ*MXAN3936* and *MXAN3938*) and with diminished prey-killing ability, did not display the inhibition zone against MG1655, consistent with earlier findings (Wang et al., 2014).

Subsequently, a comprehensive screening of all strains within the *E. coli* Large-scale Chromosome Deletion Library (NBRP) was conducted to reveal any potential molecular constituents of *E. coli* involved in the predator-prey interaction. Among these strains, an *E. coli* mutant denoted as ME5012 was identified to possess the most significantly reduced sensitivity to predation by DK1622 showing the substantially diminished inhibition zone (Figure 1A-left panel). This observation was further corroborated through the colony invasion assay and quantification of the surviving *E. coli* by CFU counting (Figure 1B).

### The identification of pivotal genes in ME5012 accountable for the reduced susceptibility to *M. xanthus* predation

**FIGURE 2:**
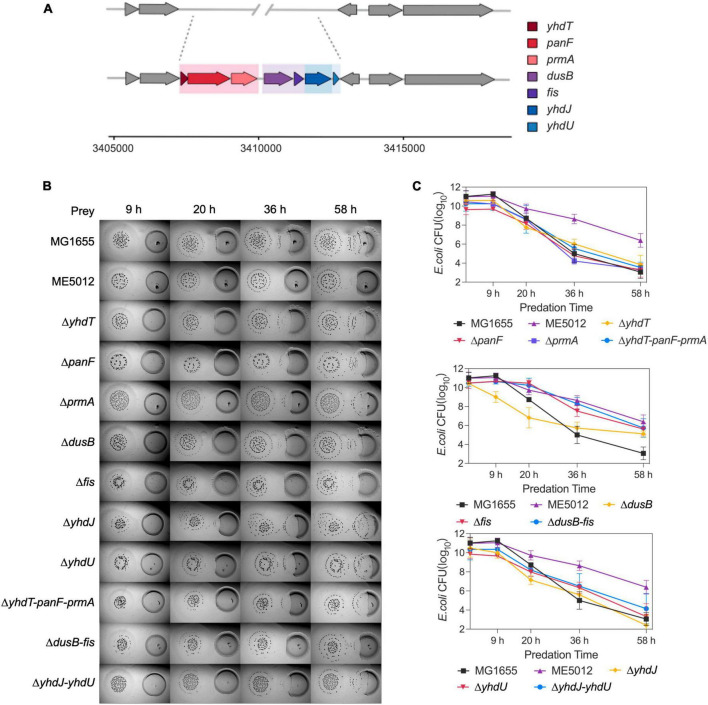
The *dusB* and *fis* genes were responsible for the less predation-susceptible phenotypes in ME5012. **(A)** ME5012 is a large-sequence-deletion *E. coli* mutant, involving seven genes, i.e., *yhdT*, *panF*, *prmA*, *dusB*, *fis*, *yhdJ*, and *yhdU*. **(B)** Visualization of colony invasion of *M. xanthus* DK1622 (left) into *E. coli* mutants (right) of all genes covered by ME5012. **(C)** Quantitative analysis of surviving *E. coli* cells during the colony invasion assay [panel **(B)**] facilitated by CFU determination at set intervals.

The deletion fragment located within ME5012 encompasses a cluster of seven genes situated at 73.39 min on the *E. coli* chromosome (Figure 2A). To elucidate the specific genes responsible for *E. coli*’s reduced sensitivity to *M. xanthus* predation within this genomic region, we systematically generated in-frame deletion mutants for each of the genes encompassed by ME5012.

As illustrated in the colony invasion assay presented in Figure 2B, and further validated through CFU quantification (Figure 2C), deletions of the *yhdT*, *panF*, *prmA*, *yhdJ*, and *yhdU* genes did not result in any noticeable alteration in the susceptibility of *E. coli* cells to predation by *M. xanthus* DK1622, which remained comparable to that of the MG1655. In contrast, the deletion of the *dusB* and *fis* genes led to a notable resistant phenotype, and simultaneous deletion of *dusB* and *fis* genes replicated the predation susceptibility observed in ME5012 (Figure 2 and Supplementary Figure 1). The complemented strains of Δ*dusB*, Δ*fis* and Δ*fis-dusB* exhibited a restoration of susceptibility to the WT MG1655 level (Supplementary Figure 2). Conversely, the deletion combinations of *yhdT*-*panF*-*prmA* or *yhdJ*-*yhdU* had no discernible impact on sensitivity. These results strongly suggest that the *dusB* and *fis* genes are pivotal in driving the observed phenotypic variations exhibited by mutant ME5012.

### Quantitative proteomics analysis of *E. coli* ME5012 compared to MG1655

**FIGURE 3:**
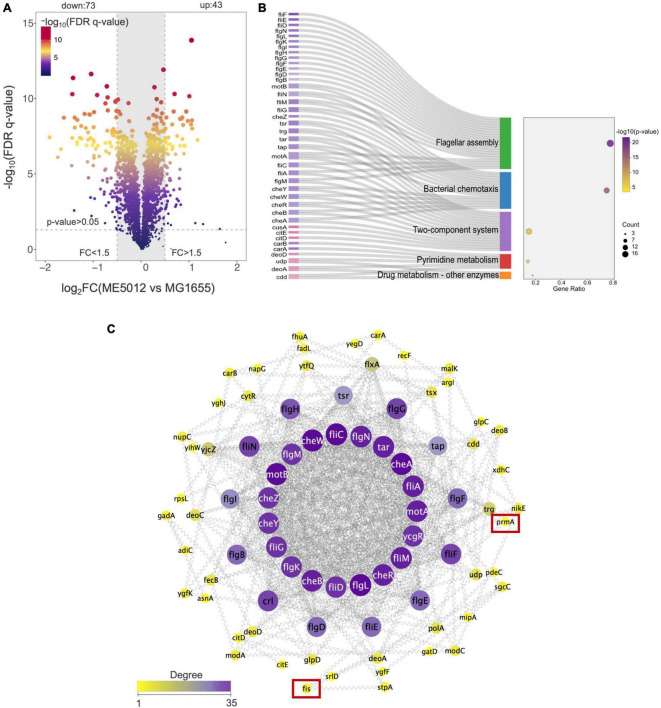
Quantitative proteomics analysis of *E. coli* ME5012 compared to MG1655. **(A)** Identification of significantly differentially expressed proteins (DEPs) in ME5012 compared to MG1655. The *X*-axis represents protein difference (log2-transformed fold changes), and the *Y*-axis represents the corresponding log10 transformed FRD *q*-value. Two vertical lines indicate expression fold change (ME5012 vs. MG1655) >1.5 and <-1.5, respectively, and a horizontal line indicates the adjusted *p*-value (FDR *q*-value) of 0.05. The *p*-values were calculated by a two-sided Wilcoxon rank-sum test, the bigger size of the dot infers a smaller FDR *q*-value, and the color of the dot represents the FDR *q*-value levels. The gray panel infers annotated proteins with insignificant differences. There are 43 dots identified as the significantly upregulated proteins, and 73 dots identified as the significantly downregulated proteins in the plot. **(B)** KEGG pathway enrichment of DEPs. The Sankey-dot plot diagram was employed to visualize top 5 enriched KEGG pathways of DEPs (*p*-value < 0.05). Related genes were listed on the left side of the panel (the pink color refers to upregulated genes, and the purple color refers to downregulated genes). The bubble diagram panel represents fold enrichment, gene count, and *p*-value KEGG pathways. The size of the dot is directly proportional to the gene count. **(C)** The protein-protein interaction (PPI) network construction based on degree centrality analysis. The node sizes represent the degree of centrality, meaning nodes with more interactions appear larger. Purple color refers to a high degree of centrality (>26).

Through literature review, we have noticed that in *E. coli*, the *dusB*-*fis* operon encodes the global regulator Fis (factor for inversion stimulation), an abundant nucleoid-associated protein (NAP) that undergoes transient expression during the early exponential phase, which serves as an important global regulator of cellular metabolism, coordinating chromosomal DNA topology and ribosomal biosynthesis (Crozat et al., 2010; Gerganova et al., 2015; Kasho et al., 2023). Given that the *dusB-fis* operon regulates a multitude of crucial physiological processes in *E. coli*, establishing a direct causal link between the mutation of the *dusB-fis* operon and the observed defensive phenotype in predation through bioinformatics analysis becomes challenging. Therefore, we conducted a quantitative proteomics analysis of ME5012 in comparison with MG1655. The iTRAQ was used to analyze total cellular proteins obtained from *E. coli* cells. As a result, 22355 peptides and 2358 proteins were identified (filter criteria = 1% FRD). FC > 1.5 and FDR *q*-value < 0.05 were used as the screening criteria for DEPs. Compared with MG1655, a total of 116 proteins were differentially expressed in the ME5012 (Figure 3A and Supplementary Table 2). Among these DEPs, 43 were upregulated and 73 were downregulated. Then, we conducted the KEGG pathway enrichment analysis to further characterize the biological functions of the identified DEPs (Supplementary Table 3). Five pathways were significantly enriched based on the *p*-value (Figure 3B), including flagella assembly (ko02040), bacterial chemotaxis (ko02030), two-component systems (ko02020), pyrimidine metabolism (ko00240), and drug metabolism-other enzymes (ko00983). Among these, the most enriched was the flagella assembly pathway including 21 downregulated genes.

### The deletion of *fis* significantly reduces flagellum production in *E. coli*, leading to a certain degree of reduced susceptibility to *M. xanthus* predation

Based on the results of proteomic analysis, we initially focused on the flagellar production in strain ME5012. The PPI network of the identified DEPs for flagella assembly was constructed as previously described (Jeong et al., 2001), and the selection standards were based on topological algorithms, including degree, betweenness centrality, and closeness centrality, ensuring a comprehensive and robust selection of hub genes. As shown in Figure 3C, among the seven genes missing in ME5012, only *fis* and *prmA* were identified as potentially part of the regulatory network involved in *E. coli* flagellar synthesis, albeit in relatively peripheral roles.

**FIGURE 4:**
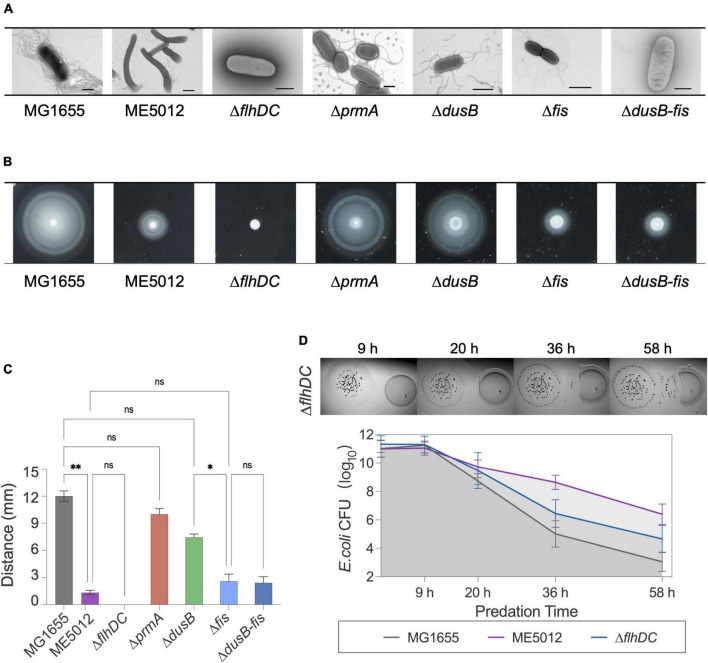
Flagellum-related phenotypes of *E. coli* strains. **(A)** Flagellar production of *E. coli* strains was observed through TEM. Scale bars are 1 μm for MG1655, 2 μm for ME5012, 1 μm for Δ*flhDC*, 2 μm for Δ*prmA*, 1 μm for Δ*dusB*, 1 μm for Δ*fis*, and 0.8 μm for Δ*dusB-fis*, respectively. **(B)** Motilities of *E. coli* strains in 0.2% agar. *E. coli* cells were spotted on LB plates with 0.2% agar and incubated at 37°C for 5 h, and the representative motility halos are shown. **(C)** The quantification of mobility halos in panel **(B)**. Error bars infer calculated standard deviations based on three replicates (*P < 0.05, **P < 0.01, ns > 0.05). **(D)** Visualization of colony invasion of *M. xanthus* DK1622 (left) into *E. coli* Δ*flhDC* mutant (right), and the quantitative analysis of surviving *E. coli* cells by CFU determination.

Next, TEM observations of mutants (Figure 4A) revealed that MG1655 exhibited abundant flagellar structures on its surface, whereas flagellar was largely absent in ME5012, consistently echoing the KEGG analysis of the limited flagella assembly pathway (Figure 3B). The control strain Δ*flhDC* (lacking essential flagellar regulatory gene *flhDC*) displayed a complete absence of flagella, which is consistent with previous report (Zhao et al., 2007). The Δ*prmA* and Δ*dusB* mutant strains continued to produce a relatively large number of flagella, while the Δ*fis* and Δ*fis-dusB* mutants nearly approached the flagellar abundance of ME5012. These observations were further confirmed through a motility assay conducted in semi-solid agar (Figures 4B, C). A marginal reduction in motility halos was noted in the Δ*prmA* and Δ*dusB* mutants compared to the WT. In contrast, both Δ*fis* and Δ*fis-dusB* strains exhibited significantly reduced motility compared to the WT, similar to the ME5012. However, their motility did not reach the level of complete loss observed in the Δ*flhDC* mutant. Subsequently, we assessed the predation sensitivity of Δ*flhDC* mutant (Figure 4D). As expected, this strain displayed some level of reduced predation susceptibility, albeit higher than that of ME5012. These results suggest a potential role for flagellar structures of *E. coli* during predation by *M. xanthus*.

**FIGURE 5:**
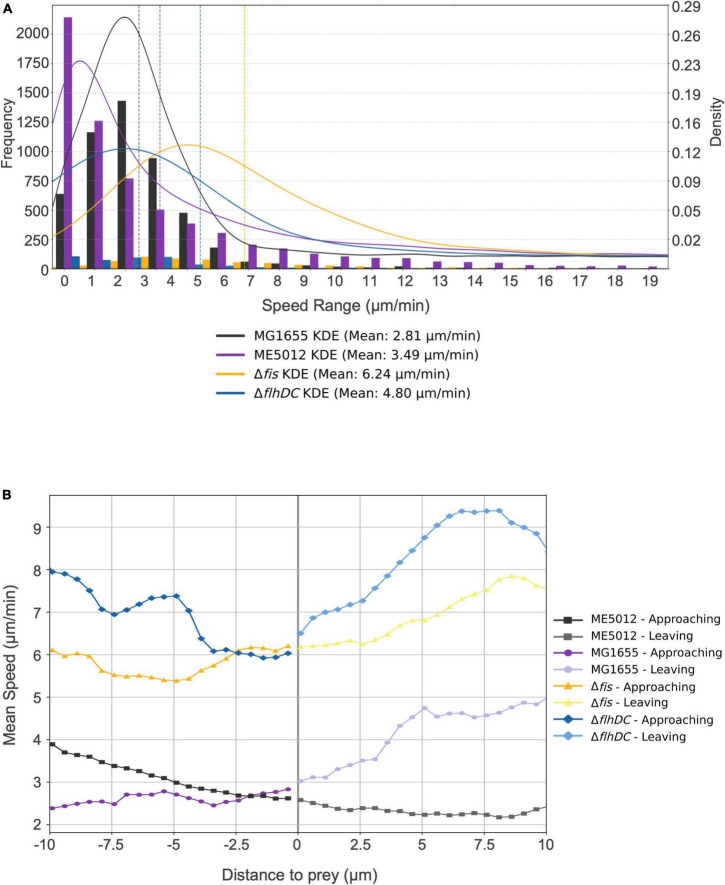
Motility characterization of *M. xanthus* DK1622 with the presence of various *E. coli* prey strains. **(A)** Frequency distribution of *M. xanthus* DK1622 cell movement speed with Kernel Density Estimation (KED) and mead speed. The set of four juxtaposed bar charts illustrates the frequency distribution of motility speeds of DK1622 cells mixed with different live *E. coli* prey cells, e.g., MG1655, ME5012, Δ*flhDC*, and Δ*fis*, respectively. The vertical dashed lines indicate the mean speed for each dataset. **(B)** The representative profile of the instantaneous speed of a *M. xanthus* DK1622 cell approaching and leaving a live *E. coli* prey cell. The *X*-axis represents the distance of a DK1622 cell to a prey cell, with negative values indicating the predator’s approach to the prey, and positive values indicating the predator’s leaving away from the prey. The zero point signifies the cell-cell contact between the predator and prey. The *Y*-axis represents the instantaneous velocity of the DK1622 cell.

To further validate this hypothesis, we employed tracking software to automatically record and analyze the movement of *M. xanthus* DK1622 during predation on various live *E. coli* prey cells. The analysis encompassed approximately 12,000 predator cells, and Kernel Density Estimation (KDE) curves were generated from the density functions of each dataset, yielding a smoothed estimate of the speed distribution for each scenario (Figure 5A). Simultaneously, we extracted and presented the motion patterns of representative individual DK1622 cells encountering different individual *E. coli* cells (Figure 5B). The results corroborate that, in the presence of live WT *E. coli* MG1655, DK1622 exhibits predatory behavior, characterized by the lowest average velocity and the speed distribution (Figure 5A), which aligns with prior observations (Zhang W. et al., 2020). Notably, the instantaneous speed of DK1622 cell significantly diminishes when departing from MG1655 cell (Figure 5B). When DK1622 encounters Δ*fis* or Δ*flhDC* mutant cells, the average velocity was markedly higher than when encountering MG1655, and a significantly increased instantaneous velocity was recorded when departing from flagellum-deficient *E. coli* cells. Intriguingly, despite ME5012 having flagellar production similar to that of Δ*fis* strain, DK1622 displayed a much lower average velocity when interacting with ME5012 compared to Δ*fis* cells. Additionally, a significantly increased instantaneous velocity was observed when DK1622 departed from ME5012 cells.

### The deletion of *dusB* leads to a diminished inducibility of *M. xanthus* TA production and a marginal decrease in susceptibility to TA

**FIGURE 6:**
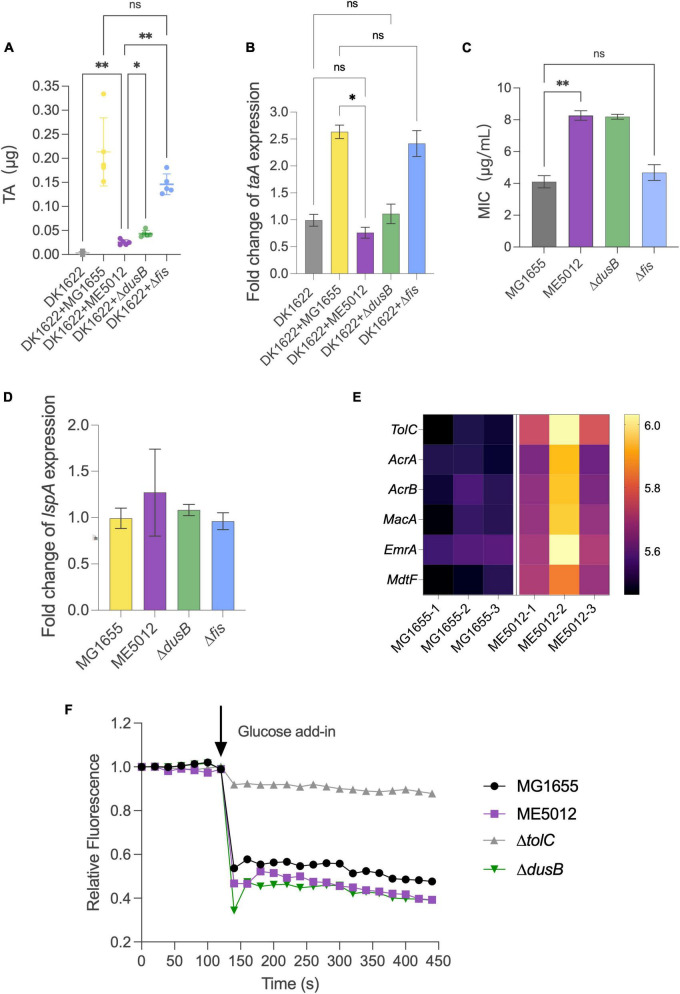
Inducibility of *M. xanthus* TA production and TA susceptibility of *E. coli* strains. **(A)** TA production by *M. xanthus* DK1622 with the presence of various *E. coli* prey strains was quantified by HPLC. **(B)** The expression of *taA* gene in DK1622 was quantified by qPCR with the presence of various E. coli prey strains. **(C)** MIC values of E. coli strains against TA (*P < 0.05, **P < 0.01, ns > 0.05). **(D)** Relative level of *lspA* gene expression in the *E. coli* mutants compared to that of MG1655. **(E)** Heatmap of some efflux pump proteins in MG1655 and ME5012 determined by the quantitative proteomic analysis. **(F)** Efflux activities in *E. coli* cells. Energy-depleted cells were loaded with Nile red and re-energized with glucose (arrow, 120 s), and efflux was measured as the decrease in Nile red fluorescence.

It has been previously established that antibiotic TA plays a pivotal role in the inhibition effects when *M. xanthus* preys on live *E. coli* (Xiao et al., 2011). The TA production of *M. xanthus* DK1622 with the presence of various live *E. coli* prey strains was quantified by HPLC. Figure 6A demonstrates that when encountering MG1655 cells, DK1622 significantly boosts the secretion of TA, thereby ensuring effective predation. However, this inductive response diminishes considerably when ME5012 and Δ*dusB* cells are used as prey. Strain Δ*fis* exhibits a significantly higher inductive capability than ME5012 and closely resembles the WT strain. To confirm this observation, additional transcriptional analyses of the TA operon were conducted. As the structural gene at the beginning of the TA operon, *taA* was selected for qPCR analysis. As shown in Figure 6B, the expression of *taA* was up-regulated approximately 2.3 to 2.6-fold when *M. xanthus* cells were, respectively, mixed with MG1655 and Δ*fis* cells, while ME5012 and Δ*dusB* failed to induce *taA* expression.

Beyond its reduced capacity to induce *M. xanthus* TA production, the mutants less susceptible to the predation may also exhibit enhanced resistance against TA lethality. To investigate this, we determined the MICs of purified TA against four *E. coli* strains (Figure 6C). ME5012 and Δ*dusB* displayed a MIC of 8 μg/mL, slightly higher than that of MG1655 and Δ*fis* (4 μg/mL). Previous reports have suggested that some prey cells increase *lspA* expression to acquire resistance to TA (Xiao et al., 2012; Wang et al., 2019). However, qPCR analysis revealed that all *E. coli* mutants exhibited similar *lspA* expression levels compared to WT MG1655 (Figure 6D). Furthermore, a re-analysis of our proteomic data unveiled the upregulated expression of some efflux pump proteins in ME5012 compared to MG1655 (Figure 6E), which has been shown to provide resistance to a variety of antimicrobial agents in *E. coli* (Borges-Walmsley and Walmsley, 2001; Kobayashi et al., 2001; Nagakubo et al., 2002). In Gram-negative bacteria, series of efflux pump systems rely on TolC to facilitate the translocation of a myriad of substrates, and the genetic ablation of *tolC* markedly attenuates efflux activity and increases in antibiotic susceptibility, while overexpression of *tolC* have been verified to directly proportional to antibiotic resistance (Cunrath et al., 2019). The Nile-red assay was employed to evaluate the efflux activities of *E. coli* cells, which is especially suitable for comparing efflux in diverse isolates (Bohnert et al., 2010; Cunrath et al., 2019). As shown in Figure 6F, MG1655 showed rapid energy-dependent efflux, whereas deletion of *tolC* abolished efflux in WT strain, which is consistent with previous observations (Bohnert et al., 2010; Cunrath et al., 2019). In addition to the representative fluorescence curves in Nile-red assay (Figure 6F), the statistical analysis of percentage fluorescence decreases and efflux half-times (Supplementary Figure 3) demonstrated that both ME5012 and Δ*dusB* strains exhibited slightly higher levels of active efflux activity compared to MG1655. These findings collectively suggest that the deletion of *dusB* in *E. coli* results in reduced inducibility of TA production by *M. xanthus*, and a slight increase in resistance to TA likely due to the overexpression of efflux pumps.

## Discussion

Microbial predator-prey relationships have traditionally emphasized the role of the predator, but recent research has illuminated the molecular responses of various prey organisms when confronted with predation by *M. xanthus*, which has revealed that a relatively limited number of overarching resistant strategies are employed by the diversified prey species (Sydney et al., 2021). While considerable attention has been directed toward studying the interaction between *M. xanthus* and *E. coli*, many aspects of *E. coli*’s response and defense mechanisms against predation by *M. xanthus* remain poorly understood, particularly considering that transcriptomic analysis has unveiled significant differential expression in approximately 40% of *E. coli* genes during co-incubation with *M. xanthus* (Livingstone et al., 2018). This knowledge gap persists, even in the face of extensive research, which has substantiated well-documented phenomena such as biofilm formation (DePas et al., 2014), upregulation of OmpT and mucoidy development (Nair et al., 2019), and overexpression of SPase II (Xiao et al., 2012). In the endeavor to identify genes in *Pseudomonas aeruginosa* that contribute to its susceptibility to *M. xanthus* predation, a cluster of prey-related proteins has emerged, suggesting that its capacity to withstand predation relies on the effectiveness of metal/oxidative stress system, motility system, and mechanisms for detoxifying antimicrobial peptides (Sydney et al., 2021). This discovery underscores the potency of whole-genome screening as a robust methodology for elucidating predation resistance mechanisms. Therefore, we conducted a comprehensive screening of the *E. coli* large-scale chromosome deletion library, culminating in the identification of the mutant ME5012, which displayed remarkably reduced susceptibility to predation by *M. xanthus*. Subsequent investigation revealed the pivotal roles played by the *dusB* and *fis* genes in driving this observed phenotype. To the best of our knowledge, this represents the initial report detailing the involvement of the global regulator encoded by the *dusB-fis* operon (Crozat et al., 2010; Gerganova et al., 2015; Kasho et al., 2023) in the regulation of prey susceptibility in *E. coli*.

Fis is a highly abundant small DNA-binding protein within *E. coli*, playing a multifaceted role in regulating various biological processes, including transcription, recombination, replication reaction, nucleoid compaction, and chromosome conformation (Crozat et al., 2010; Nafissi et al., 2012; Duprey et al., 2014; Kasho et al., 2023). The expression profile of Fis is characterized by elevated levels during rapid growth under nutrient-rich conditions, while it diminishes during slow growth in nutrient-poor environments or in the stationary phase (Ali Azam et al., 1999). Positioned upstream of *fis* gene within the operon, the *dusB* gene encodes a tRNA synthase in *E. coli*, responsible for modifying tRNAs by converting uridine to 5,6-dihydrouridine within D loops (Bishop et al., 2002). The regulatory control of Fis synthesis is primarily exercised at the transcription initiation site of the *dusB*-*fis* operon (Cheng et al., 2000), and although translation of *dusB* is limited, it plays a key role in facilitating the robust translation of *fis* (Crozat et al., 2010; Nafissi et al., 2012; Burkhardt et al., 2017). Furthermore, the analyses of several mutations affecting the transcription, translation, and protein activity of Fis in both *fis* and *dusB* genes have demonstrated substantial parallelism in the resulting phenotypic effects (Crozat et al., 2010). Our findings indicate that while the individual deletion of *dusB* and *fis* genes confers notable reduction in the sensitivity of *E. coli* to predation by *M. xanthus*, only the simultaneous deletion of both *dusB* and *fis* genes replicates the predation susceptibility observed in ME5012. This suggests that these two genes may play non-identical roles in the anti-predation process.

The quantitative proteomics analysis of ME5012 revealed a conspicuous alteration in the flagella assembly pathway, with 21 related genes showing significant downregulation compared to MG1655. In line with these findings, ME5012 exhibited markedly reduced motility in semi-solid agar compared to the WT, and a notable deficiency in flagellar production was observed through TEM. Strikingly, only the Δ*fis* and Δ*fis-dusB* strains displayed a significant reduction in motility, akin to the ME5012 strain. The Δ*fis* mutant nearly reached the flagellar production level of ME5012, while the Δ*dusB* mutant exhibited only a slight reduction in flagellar synthesis compared to the WT. Our observations align with previous reports, e.g., the *fis*:Km mutant of adherent-invasive *E. coli* (AIEC) failing to express flagellin (Miquel et al., 2010), implying a potential regulatory role of Fis in *E. coli* flagellar synthesis. In a Gram-negative plant pathogen *Dickeya dadantii* (formerly *Erwinia chrysanthemi*), Fis plays an essential role in the expression of the main virulence genes, and binds to the promoter regions of *fliC* to activate *fli* operon expression and flagella production (Lautier and Nasser, 2007; Duprey et al., 2014). Furthermore, the Δ*flhDC* mutant abolishing flagella production exhibited lower degree of predation susceptibility, albeit lower than that of ME5012, hinting at the possible involvement of *E. coli*’s flagellar structures in predation by *M. xanthus*. The analysis of *M. xanthus* DK1622 cell movement suggested that surface flagella on WT *E. coli* cells might be recognized by *M. xanthus* during the solitary predation, potentially trapping the predator cells and reducing their motility velocity, thus hindering their departure from the prey. Conversely, in cases where flagella were absent, as seen in the Δ*flhDC* mutant, DK1622 cells exhibited significantly higher movement speeds, diminishing their chances of contacting prey and reducing predation efficiency. Therefore, the deletion of the *fis* gene substantially curtails flagellum production in *E. coli*, resulting in a certain level of reduced susceptibility to *M. xanthus* predation. However, the distinct motility patterns observed in DK1622 cells when encountering ME5012 and the Δ*fis* mutant suggest a more intricate mechanism underpinning ME5012’s predation resistance, despite the similarities in predation susceptibility and flagella production between ME5012 and the Δ*fis* mutant.

It is particularly interesting to note the up-regulation of TA production and the *taA* gene of DK1622 in response to direct contact with the specific live *E. coli* prey. When *M. xanthus* encounters MG1655 cells, there is a significant augmentation in TA secretion, thereby enhancing predation efficacy. This finding lends support to previous research indicating that antibiotic TA plays a pivotal role in the inhibition/killing effects when *M. xanthus* preys on live *E. coli* (Xiao et al., 2011). The inability to elicit the up-regulation of *M. xanthus* TA production by ME5012 suggests the requirement for specific genetic components in *E. coli*. Indeed, this inductive response diminishes significantly when Δ*dusB* cells are employed as prey, in contrast to Δ*fis* cells. However, the mechanism underlying this observed phenotype remains elusive, and the question of how *M. xanthus* can discern the presence of different prey and respond differentially presents an intriguing avenue for further exploration. Furthermore, both ME5012 and Δ*dusB* strains exhibited a MIC slightly higher than that of MG1655 and Δ*fis*, which cannot be attributed to the overexpression of *lspA*. Proteomic analysis revealed the up-regulation of several efflux pump proteins in ME5012 compared to MG1655. Both ME5012 and Δ*dusB* strains displayed slightly higher levels of active efflux activity than MG1655, which might contribute to their slight increase in resistance against TA. Nevertheless, establishing a direct connection between DusB and efflux pumps warrants further investigation.

In conclusion, our investigation sheds light on the complex interplay of genetic components within *E. coli* during its interaction with *M. xanthus* in a predator-prey context. The prominent parts of the *dusB* and *fis* genes in driving a reduced predation-susceptible phenotype underscore the multifaceted nature of the defense mechanisms employed by *E. coli*. Although the roles of these genes in governing flagellum production and responses related to TA have been proposed, the intricacies concerning the functions of the *dusB-fis* operon in *E. coli*’s reaction to *M. xanthus*’s tad-like (Seef et al., 2021) and type III-like (Thiery et al., 2022) apparatus-mediated contact-dependent killing require more in-depth examinations. Furthermore, our findings prompt further investigations into the nuances surrounding the functions of the *dusB-fis* operon in *E. coli*’s response to non-antibiotic substances, which also play an essential role in *M. xanthus* prey killing (Zwarycz et al., 2020), particularly in light of their potential facilitation by OMVs (Zwarycz et al., 2023). Unraveling the mechanisms underlying the interactions between *M. xanthus* and *E. coli* will provide deeper insights into the predator-prey dynamics and the broader ecological implications of such interactions.

## Data availability statement

The data presented in the study are deposited in the ProteomeXchange Consortium via the PRODE partner repository with accession number PXD046017.

## Author contributions

NZ: Conceptualization, Writing – original draft, Writing – review and editing, Data curation, Investigation, Methodology, Software, Validation, Visualization. TL: Data curation, Writing – review and editing. HP: Methodology, Writing – review and editing, Conceptualization, Data curation, Resources. YW: Writing – review and editing, Data curation, Methodology, Software, Visualization. QL: Writing – review and editing, Data curation. JL: Writing – review and editing, Data curation, Investigation. XH: Resources, Writing – review and editing. WS: Conceptualization, Writing – review and editing, Resources. YL: Writing – review and editing, Resources. CW: Data curation, Writing – review and editing, Writing – original draft, Funding acquisition. FZ: Writing – review and editing, Supervision, Writing – original draft, Methodology. WH: Writing – review and editing, Conceptualization, Funding acquisition, Supervision, Writing – original draft.
