# Supplementary material for: Genetic components of Escherichia coli involved in its complex prey-predator interaction with Myxococcus xanthus
Source: Front Microbiol. 2023 Dec 5;14:1304874. doi: 10.3389/fmicb.2023.1304874 (PMC10728724; doi:10.3389/fmicb.2023.1304874)
Supplement: Supplementary file 1 [file Data_Sheet_3.docx]

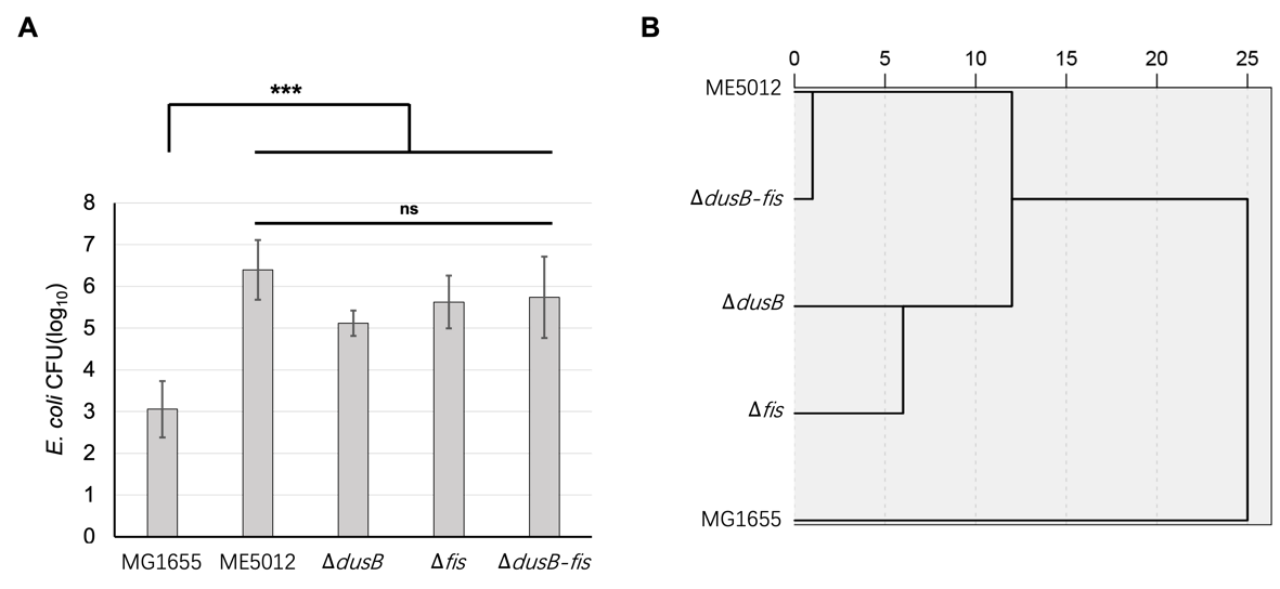


**Figure S1. Variance and hierarchical cluster analysis of survival CFU data presented in Fig. 2C.**

A. Analysis of variance (ANOVA) was employed to assess variations in survival CFUs among wild-type and mutant strains of *E. coli* following a 58-hour exposure to predation by *M. xanthus* DK1622 (* P < 0.05; ** P < 0.01; *** P < 0.001; ns > 0.05).

B. Hierarchical cluster analysis was conducted on wild-type and mutant strains based on their survival CFUs after exposure to predation by *M. xanthus* DK1622 at 9 h, 20 h, 36 h, and 58 h.


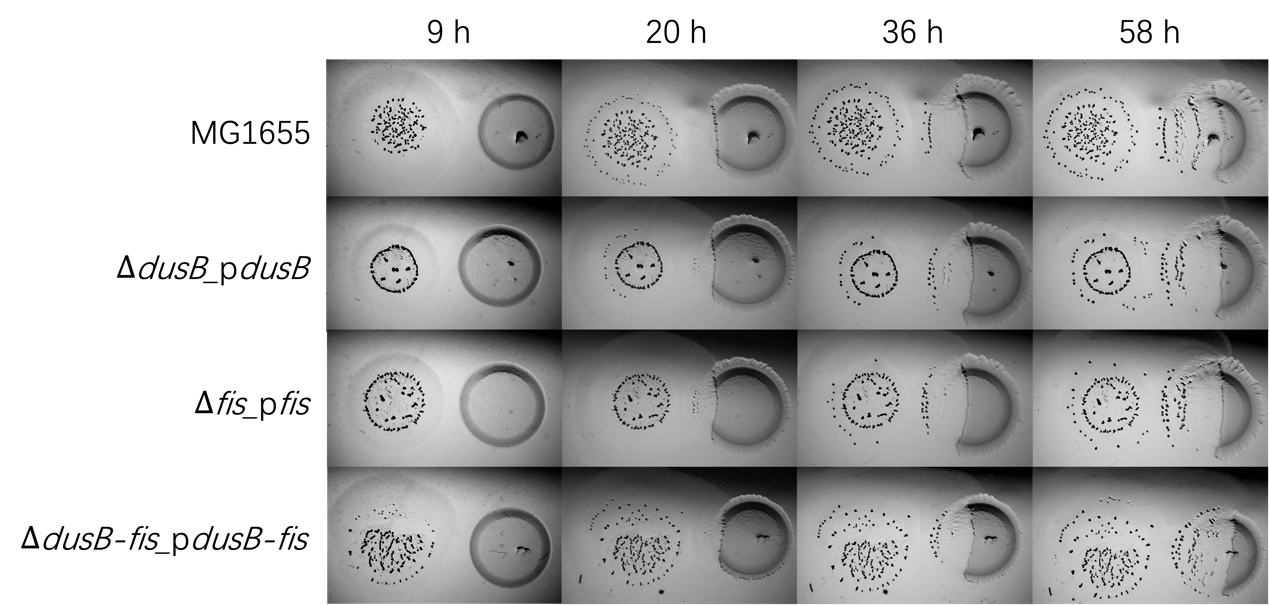


**Figure S2.** **Visualization of colony invasion of *M. xanthus* DK1622 (left) into *E. coli* complemented strains (right).**


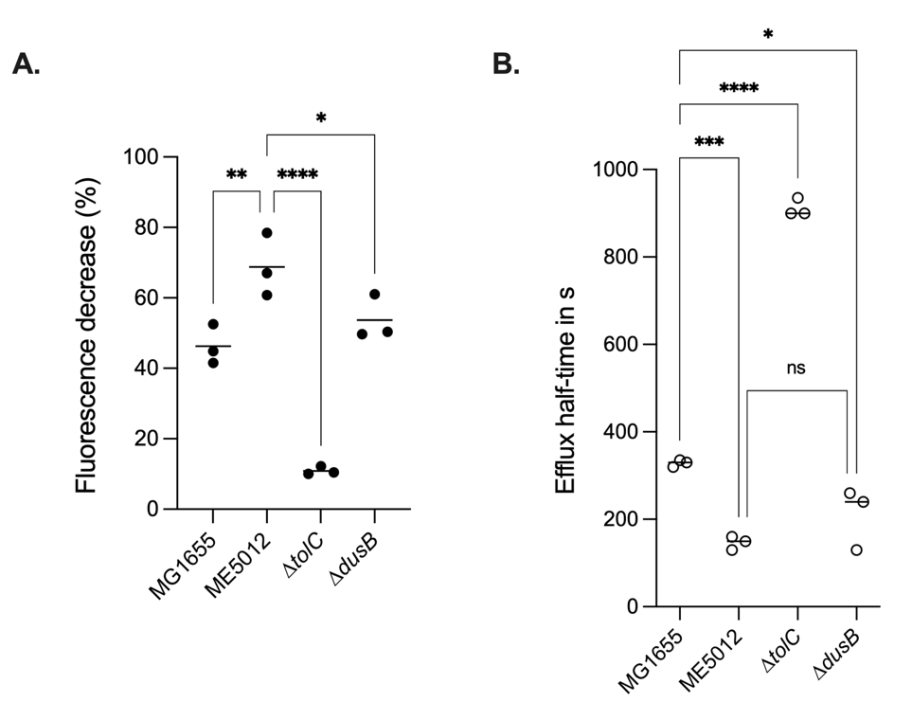


**Figure S3. Efflux activities in *E. coli* cells.**

A. Percentage fluorescence decreases in Nile-red assay. Values of 100 correspond to complete loss of Nile-red fluorescence, and higher values indicates higher efflux activity/efficiency after energization. *∆tolC* was set as a negative control. Three biological replicates were performed on each *E. coli* strains (* P < 0.05; ** P < 0.01; *** P < 0.001; ns > 0.05).

B. Efflux half-time was dotted with three replicates to visualize the time intervals of *E. coli* strains lost 50% fluorescence intensity after glucose energization. Decreased half-time intervals indicated higher efflux quality (* P < 0.05; ** P < 0.01; *** P < 0.001; ns > 0.05).
