# Supplementary material for: Genetic components of Escherichia coli involved in its complex prey-predator interaction with Myxococcus xanthus
Source: Front Microbiol. 2023 Dec 5;14:1304874. doi: 10.3389/fmicb.2023.1304874 (PMC10728724; doi:10.3389/fmicb.2023.1304874)
Supplement: Supplementary file 2 [file Table_1.DOCX]

**Supplementary Table S1. Bacterial strains, plasmids, and primers**

| **Name** | **Relevant Description** | **Source/Reference** |
| --- | --- | --- |
| **Strain** |  |  |
| *Myxococcus xanthus* |  |  |
| DK1622 | Wild type strain | Lab stock |
| YL0912 | DK1622, △*MXAN3936*, △*MXAN3938* | (Wang *et al.* 2014) |
| *Escherichia coli* |  |  |
| MG1655 | Wild type strain | Lab stock |
| ME strains | 124 *E. coli* mutant strains from the Large-scale Chromosome Deletion Library | (Kato *et al.*, 2008) |
| ME5012 | MG1655, a chromosome deletion mutant | NBRP |
| *∆yhdT* | MG1655, *∆yhdT* | This study |
| *∆panF* | MG1655, *∆panF* | This study |
| *∆prmA* | MG1655, *∆prmA* | This study |
| *∆dusB* | MG1655, *∆dusB* | This study |
| *∆fis* | MG1655, *∆fis* | This study |
| *∆yhdJ* | MG1655, *∆yhdJ* | This study |
| *∆yhdU* | MG1655, *∆yhdU* | This study |
| *∆yhdT-panF-prmA* | MG1655, *∆yhdT-panF-prmA* | This study |
| *∆dusB-fis* | MG1655, *∆dusB-fis* | This study |
| *∆yhdJ-yhdU* | MG1655, *∆yhdJ-yhdU* | This study |
| *∆dusB-fis-yhdJ-yhdU* | MG1655, *∆dusB-fis-yhdJ-yhdU* | This study |
| Δ*flhDC* | MG1655, Δ*flhDC* | This study |
| Δ*tolC* | MG1655, Δ*tolC* | This study |
| *∆dusB*_p*dusB* | MG1655, *∆dusB*, complemented by p*dusB* plasmid | This study |
| *∆fis*_p*dusB* | MG1655, *∆fis*, complemented by p*fis* plasmid | This study |
| *∆dusB-fis*_p*dusB-fis* | MG1655, *∆dusB-fis*, complemented by p*dusB-fis* plasmid | This study |
|  |  |  |
| **Plasmid** |  |  |
| pKD4 | plasmid with FRT-flanked Kan^r^ cassette | (Datsenko and Wanner 2000) |
| pTKRed | Helper plasmid with, Spe^r^ | (Zhang *et al.* 2020) |
| pCP20 | temperature-sensitive replication and thermal induction of FLP synthesis, Amp^r^_,_ and Cm^r^ | (Datsenko and Wanner 2000) |
| p*fis* | The pTrc99a vector containing MG1655 *fis* gene, Amp^r^ | (Gibson et al., 2009) |
| p*dusB* | The pTrc99a vector containing MG1655 *dusB* gene, Amp^r^ | (Gibson et al., 2009) |
| p*dusB*-*fis* | The pTrc99a vector containing MG1655 *dusB* and *fis* gene, Amp^r^ | (Gibson et al., 2009) |
| **Primer** |  |  |
| PCR Primer | 5’- 3’ |  |
| *yhdT*-P1-F | ggatcttccagagatGAAAACGGCGAGTTCTATTTCATC | This study |
| *yhdT*-P1-R | ctacacaatcgctcaaTTTTTGTCCCTGATGGGTGAAG | This study |
| *yhdT*-P2-F | gtcagccgttaagtgtAGTAATTCTACCGCTGGTCGC | This study |
| *yhdT*-P2-R | ctgccgttcgacgatGTACCCAGAACGACGTCATAAAG | This study |
| *yhdT*-R-F | CATCAGGGACAAAAAttgagcgattgtgtaggctg | This study |
| *yhdT*-R-R | CCAGCGGTAGAATTACTacacttaacggctgacatgggaattag | This study |
| *panF*-P1-F | ggatcttccagagatGGCGTACGTTGGGAGTCTCATATCT | This study |
| *panF*-P1-R | cctacacaatcgctcaaCGTCATCCTCCAGTGGGATATCG | This study |
| *panF*-P2-F | gtcagccgttaagtgtAGAGTTTTGCCATGCCTTGGATC | This study |
| *panF*-P2-R | ctgccgttcgacgatCATAAGCTTCACAAACGCTCTCTG | This study |
| *panF*-R-F | CCACTGGAGGATGACGttgagcgattgtgtaggctg | This study |
| *panF*-R-R | GGCATGGCAAAACTCTacacttaacggctgacatgggaattag | This study |
| *prmA*-P1-F | ggatcttccagagatGATCCCAACGTTAATGGTAAAAGTG | This study |
| *prmA*-P1-R | cctacacaatcgctcaaGGCAAAACTCTTTATTTATCAGTAGTC | This study |
| *prmA*-P2-F | gtcagccgttaagtgtCCTTCGCATCGCCGTAGGGT | This study |
| *prmA*-P2-R | ctgccgttcgacgatAATCTTCAGGGTAACAGGAACGTC | This study |
| *prmA*-R-F | GATAAATAAAGAGTTTTGCCttgagcgattgtgtaggctg | This study |
| *prmA*-R-R | CGGCGATGCGAAGGacacttaacggctgacatgggaattag | This study |
| *dusB*-P1-F | ggatcttccagagatGCCGATAGCTTCGCACTGGAC | This study |
| *dusB*-P1-R | cctacacaatcgctcaaGAGTTAAGAAATGACCATACTGTGACTGCAAG | This study |
| *dusB*-P2-F | gtcagccgttaagtgtACAGAAATAAAGAGCTGACAGAACTATGTTCG | This study |
| *dusB*-P2-R | ctgccgttcgacgatAACTGTCATATGACCAGACGATGCG | This study |
| *dusB*-R-F | GTATGGTCATTTCTTAACTCttgagcgattgtgtaggctg | This study |
| *dusB*-R-R | GCTCTTTATTTCTGTacacttaacggctgacatgggaattag | This study |
| *fis*-P1-F | ggatcttccagagatCAGTGGACGTTCCTGTTACCCTGAAG | This study |
| *fis*-P1-R | ccagcctacacaatcgctcaaAGTTCTGTCAGCTCTTTATTTCTGTTTACGC | This study |
| *fis*-P2-F | cccatgtcagccgttaagtgtTTCAGGTTAGCTAAATGCTTGATTAAAAAGG | This study |
| *fis*-P2-R | ctgccgttcgacgatTGATTGTAAGGCTGTGGAGGATTTTTG | This study |
| *fis*-R-F | GCGTAAACAGAAATAAAGAGCTGACAGAACTttgagcgattgtgtaggctggag | This study |
| *fis*-R-R | CAAGCATTTAGCTAACCTGAAacacttaacggctgacatgggaattag | This study |
| *yhdJ*-P1-F | ggatcttccagagatCATTATCTGGACACTGGGGAGT | This study |
| *yhdJ*-P1-R | cctacacaatcgctcaaGGTCACTCCCTTTGTGACACC | This study |
| *yhdJ*-P2-F | gtcagccgttaagtgtAGGATGTGTAAGCCTGGTTTTC | This study |
| *yhdJ*-P2-R | ctgccgttcgacgatGCTATTTTCTCACTCTGTGTCGAAT | This study |
| *yhdJ-*R-F | CACAAAGGGAGTGACCttgagcgattgtgtaggctg | This study |
| *yhdJ*-R-R | GCTTACACATCCTacacttaacggctgacatgggaattag | This study |
| *yhdU*-P1-F | ggatcttccagagatTGCCCTTTATCGATCTCCAGTG | This study |
| *yhdU*-P1-R | cctacacaatcgctcaaTACCTGAGCCTGTGCATGTTTAAC | This study |
| *yhdU*-P2-F | gtcagccgttaagtgtATTTACATGACACTTAATTCATTCGTTTGATG | This study |
| *yhdU*-P2-R | ctgccgttcgacgatGAAGACCCGGCAAGAACTGATTG | This study |
| *yhdU*-R-F | ACATGCACAGGCTCAGGTAttgagcgattgtgtaggctg | This study |
| *yhdU*-R-R | TAAGTGTCATGTAAATacacttaacggctgacatgggaattag | This study |
| *yhdT/panF/prmA*-P1-F | ggatcttccagagatGAAAACGGCGAGTTCTATTTCATC | This study |
| *yhdT/panF/prmA*-P1-R | ctacacaatcgctcaaTTTTTGTCCCTGATGGGTGAAG | This study |
| *yhdT/panF/prmA*-P2-F | gtcagccgttaagtgtCCTTCGCATCGCCGTAGGGT | This study |
| *yhdT/panF/prmA*-P2-R | ctgccgttcgacgatAATCTTCAGGGTAACAGGAACGTC | This study |
| *yhdT/panF/prmA*-R-F | GATAAATAAAGAGTTTTGCCttgagcgattgtgtaggctg | This study |
| *yhdT/panF/prmA*-R-R | CGGCGATGCGAAGGacacttaacggctgacatgggaattag | This study |
| *dusB/fis*-P1-F | ggatcttccagagatGTAATGGCGTTTCTGACCGTCTG | This study |
| *dusB/fis*-P1-R | cctacacaatcgctcaaGAGTTAAGAAATGACCATACTGTGACTGCAAG | This study |
| *dusB/fis*-P2-F | gtcagccgttaagtgtTTCAGGTTAGCTAAATGCTTG | This study |
| *dusB/fis*-P2-R | ctgccgttcgacgatTTTTGATGTACTCGCTGTTGATC | This study |
| *dusB/fis*-R-F | GTATGGTCATTTCTTAACTCttgagcgattgtgtaggctg | This study |
| *dusB/fis*-R-R | ATTTAGCTAACCTGAAacacttaacggctgacatgggaattag | This study |
| *yhdJ/yhdU*-P1-F | ggatcttccagagatATTGAGGATGCCAGCGAACA | This study |
| *yhdJ/yhdU*-P1-R | cagcctacacaatcgcGGTCACTCCCTTTGTGACACC | This study |
| *yhdJ/yhdU*-P2-F | atggaccatggctaattcccaATTTACATGACACTTAATTCATTCGTTTG | This study |
| *yhdJ/yhdU*-P2-R | ctgccgttcgacgatTCTACTGGCACTTCGAAAACA | This study |
| *yhdJ/yhdU*-R-F | GTCACAAAGGGAGTGACCgcgattgtgtaggctggag | This study |
| *yhdJ/yhdU*-R-R | AATGAATTAAGTGTCATGTAAATtgggaattagccatggtcca | This study |
| *flhDC*-P1-F | ggatcttccagagatGCTGTATGAATCCCGCCTGA | This study |
| *flhDC*-P1-R | gcctacacaatcgctcaaTATTCCCACCCAGAATAACCAAC | This study |
| *flhDC*-P2-F | gtcagccgttaagtgtCTGATACGGTGAGGCGCAAC | This study |
| *flhDC*-P2-R | ctgccgttcgacgatAAGGTGTTCATGTGACCGCT | This study |
| *flhDC*-R-F | GTTATTCTGGGTGGGAATAttgagcgattgtgtaggctg | This study |
| *flhDC*-R-R | CGCCTCACCGTATCAGacacttaacggctgacatgggaattag | This study |
| *tolC*-P1-F | ggatcttccagagatATTGACGAACGCTCACTGGT | This study |
| *tolC*-P1-R | ctacacaatcgctcaaTTGCATTCCTTGTGGTGAAGC | This study |
| *tolC*-P2-F | gtcagccgttaagtgtTGACGACGACGGGGCTTC | This study |
| *tolC*-P2-R | ctgccgttcgacgatCTGGTTTTCTGGTGCCATGC | This study |
| *tolC*-R-F | CACCACAAGGAATGCAAttgagcgattgtgtaggctg | This study |
| *tolC*-R-R | AGCCCCGTCGTCGTCAacacttaacggctgacatgggaattag | This study |
| pTrc99a-PF | CGAGAGTAGGGAACTGCCAG | This study |
| pTrc99a-PR | GGTCTGTTTCCTGTGTGAAATTG | This study |
| 99a-*fis*-PF | aacaatttcacacaggaaacagaccATGTTCGAACAACGCGTAAATTCTGAC | This study |
| 99a-*fis*-PR | agttccctactctcgGCTAACCTGAATTAGTTCATGCCGTA | This study |
| 99a-*dusB*-PF | aacaatttcacacaggaaacagaccATGCGCATCGGACAATATCAG | This study |
| 99a-*dusB*-PR | agttccctactctcgTTACGCAAAATTTTCGAAGTATGCC | This study |
| RT-qPCR Primer | 5’- 3’ |  |
| *taA-F* | GCTCAGTTGGGGAAACACGGCTACG | This study |
| *taA-R* | GAGCGGCTGGTAACGACAGAAAAGG | This study |
| *gapA*-F | AACTGAATGGCAAACTGACTGGTA | This study |
| *gapA*-R | TTTCATTTCGCCTTCAGCAGC | This study |

**Reference**

Datsenko, K. A., and B. L. Wanner. 2000. 'One-step inactivation of chromosomal genes in Escherichia coli K-12 using PCR products', *Proc Natl Acad Sci U S A*, 97: 6640-5.

Gibson, D. G., Young, L., Chuang, R. Y., Venter, J. C., et al. (2009). 'Enzymatic assembly of DNA molecules up to several hundred kilobases', *Nat Methods*, 6(5), 343-345. doi:10.1038/nmeth.1318.

Kato, J., Hashimoto, M. (2008). Construction of long chromosomal deletion mutants of *Escherichia coli* and minimization of the genome. *Methods Mol Biol* 416, 279-93. doi: 10.1007/978-1-59745-321-9_18.

Wang, Y., X. Li, W. Zhang, X. Zhou, and Y. Z. Li. 2014. 'The groEL2 gene, but not groEL1, is required for biosynthesis of the secondary metabolite myxovirescin in Myxococcus xanthus DK1622', *Microbiology (Reading)*, 160: 488-95.

Zhang, F., B. Li, H. Dong, M. Chen, S. Yao, J. Li, H. Zhang, X. Liu, H. Wang, N. Song, K. Zhang, N. Du, S. Xu, and L. Gu. 2020. 'YdiV regulates Escherichia coli ferric uptake by manipulating the DNA-binding ability of Fur in a SlyD-dependent manner', *Nucleic Acids Res*, 48: 9571-88.
